# Could venous thromboembolism and major bleeding be indicators of lung cancer mortality? A nationwide database study

**DOI:** 10.1186/s12885-020-06930-1

**Published:** 2020-05-24

**Authors:** Jennifer Howlett, Eric Benzenine, Jonathan Cottenet, Pascal Foucher, Philippe Fagnoni, Catherine Quantin

**Affiliations:** 1 CHRU Dijon, Pharmacy, F-21000 Dijon, France; 2 Biostatistics and Bioinformatics (DIM), University Hospital, Bourgogne Franche-Comté University, Dijon, France; 3 INSERM, CIC 1432, Clinical Investigation Center, clinical epidemiology/ clinical trials unit, Dijon University Hospital, Dijon, France; 4 Department of Thoracic Oncology CHU, Dijon, France; 5 Unité INSERM U866, Dijon University Hospital, Dijon, France; 6 Biostatistics, Biomathematics, Pharmacoepidemiology and Infectious Diseases (B2PHI), INSERM, UVSQ, Institut Pasteur, Université Paris-Saclay, Paris, France

**Keywords:** Lung cancer, Venous thromboembolism, Anticoagulant therapy, Major bleeding, Medico-administrative data

## Abstract

**Background:**

Venous thromboembolism (VTE) is highly prevalent in cancer patients and can cause severe morbidity. VTE treatment is essential, but anticoagulation increases the risk of major bleeding. The purpose was to evaluate the impact of VTE and major bleeding on survival and to identify significant risk factors for these events in lung cancer patients.

**Methods:**

Data were extracted from a permanent sample of the French national health information system (including hospital and out-of-hospital care) from 2009 to 2016. All episodes of VTE and major bleeding events within one year after cancer diagnosis were identified. A Cox model was used to analyse the effect of VTE and major bleeding on the patients’ one-year survival. VTE and major bleeding risk factors were analysed with a Fine and Gray survival model.

**Results:**

Among the 2553 included patients with lung cancer, 208 (8%) had a VTE episode in the year following diagnosis and 341 (13%) had major bleeding. Almost half of the patients died during follow-up. Fifty-six (60%) of the patients presenting with pulmonary embolism (PE) died, 48 (42%) of the patients presenting with deep vein thrombosis (DVT) alone died and 186 (55%) of those presenting with a major bleeding event died. The risk of death was significantly increased following PE and major bleeding events. VTE concomitant with cancer diagnosis was associated with an increased risk of VTE recurrence beyond 6 months after the first VTE event (sHR = 4.07 95% CI: 1.57–10.52). Most major bleeding events did not appear to be related to treatment.

**Conclusion:**

VTE is frequent after a diagnosis of lung cancer, but so are major bleeding events. Both PE and major bleeding are associated with an increased risk of death and could be indicators of lung cancer mortality.

## Background

Venous thromboembolism (VTE) is a condition which includes both deep vein thrombosis (DVT) and pulmonary embolism (PE). Its annual prevalence in France has been estimated at around 180 cases (120 DVT and 60 PE) per 100,000 inhabitants [1, 2]. VTE is often asymptomatic, but the risk of recurrence and complications is high [3]. Even when it is not symptomatic, VTE is associated with increased mortality [4]. Despite clear guidelines regarding prevention and treatment, the majority of VTE cases treated in French hospitals occur during the hospital stay [5].

**Table 1:** Patient demographics and clinical characteristics and probability of VTE and major bleeding

		N (%)	VTE	PE	Bleeding
Total		2553	208 (8.21)	93 (3.6)	341 (13.4)
Age	< 55	398 (15.6)	45 (11.3)	18 (4.5)	42 (10.8)
	55–65	724 (28.4)	73 (10.1)	27 (3.7)	83 (11.5)
	65–75	703 (27.5)	54 (7.7)	24 (3.4)	103 (14.7)
	≥ 75	728 (28.5)	36 (5.0)	24 (3.3)	112 (15.4)
Gender	Male	1765 (69.1)	141 (8.0)	54 (3.1)	260 (14.7)
	Female	788 (30.9)	67 (8.5)	39 (4.9)	81 (10.3)
Charlson’s comorbidity Index	≤ 5	1811 (70.9)	159 (8.8)	37 (2.7)	239 (13.2)
	> 5	742 (29.1)	49 (6.6)	56 (4.8)	102 (13.8)
Metastatic	No	1018 (39.9)	41 (4.0)	16 (1.6)	125 (12.3)
	Yes	1535 (60.1)	167 (10.9)	77 (5.0)	216 (14.1)
VTE concomitant with cancer diagnosis	No	2489 (97.5)	201 (8.1)	90 (3.6)	329 (13.2)
	Yes	64 (2.5)	7 (10.9)	3 (4.7)	12 (18.8)
Anticoagulant or antiplatelet treatment	No	1706 (66.8)	158 (76.0)	71 (76.3)	199 (58.4)
	Yes	847 (33.2)	50 (24.0)	22 (23.7)	142 (41.6)
Anticoagulant therapy	No	2338 (91.6)	195 (8.3)	86 (3.7)	293 (12.5)
	Yes	215 (8.4)	13 (6.1)	7 (3.3)	48 (22.3)
Antiplatelet therapy	No	1851 (72.5)	168 (9.1)	78 (4.2)	227 (12.3)
	Yes	702 (27.5)	40 (5.7)	15 (2.1)	114 (16.2)
NSAID therapy	No	2255 (88.3)	175 (7.8)	80 (3.6)	311 (13.8)
	Yes	298 (11.7)	33 (11.1)	13 (4.4)	30 (10.1)

**Table 2:** One-year survival analysis after lung cancer diagnosis

		Univariate		Multivariate*	
		HR (95% CI)	*p*-value	HR (95% CI)	p-value
Age	< 55	–	–	–	–
	55–65	0.91 (0.75–1.10)	0.341	0.93 (0.77–1.26)	0.450
	65–75	1.00 (0.83–1.21)	0.988	1.05 (0.87–1.27)	0.609
	≥ 75	1.75 (1.46–2.09)	< 0.0001	2.02 (1.69–2.42)	< 0.0001
Gender	Male	–	–	–	–
	Female	0.89 (0.78–1.01)	0.061	0.85 (0.75–0.97)	0.013
Metastasis		3.46 (2.77–4.32)	< 0.0001	4.39 (3.83–5.03)	< 0.0001
PE after cancer diagnosis		2.43 (1.94–3.06)	< 0.0001	1.53 (1.20–1.95)	0.0006
DVT after cancer diagnosis		1.60 (1.29–1.97)	< 0.0001	1.26 (1.01–1.57)	0.043
Major bleeding		2.17 (1.86–2.54)	< 0.0001	1.81 (1.54–2.12)	< 0.0001

*Mutivariate analysis adjusted for age and gender

**Table 3:** Treatments and hospitalizations for patients within 1 year after lung cancer diagnosis

N (%)		Total (*N* = 2553)	Before VTE (*N* = 208)	Before PE (*N* = 93)	Before bleeding (*N* = 341)
Lung cancer surgery	No	2052 (80.4)	181 (87.0)	83 (89.3)	280 (82.1)
	Yes	501 (19.6)	27 (13.0*)	10 (10.8)	61 (17.9)
Other surgery	No	1970 (77.2)	156 (75.0)	73 (78.5)	279 (81.8)
	Yes	583 (22.8)	52 (25.0)	20 (21.5)	62 (18.2)
Chemotherapy	No	1201 (47.0)	72 (34.6)	35 (37.6)	217 (63.6)
	Yes	1352 (53.0)	136 (65.4)	58 (62.4)	124 (36.4)
+ Bevacizumab	No	1244 (92.0)	125 (91.9)	55 (91.4)	117 (94.4)
	Yes	108 (8.0)	11 (8.1)	3 (8.6)	7 (5.7)
Oral lung cancer targeted therapy	No	2340 (91.7)	201 (96.6)	87 (93.6)	333 (97.7)
	Yes	213 (8.3)	7 (3.4)	6 (6.5)	8 (2.4)
Radiotherapy	No	1711 (67.0)	155 (74.5)	68 (73.1)	274 (80.4)
	Yes	842 (33.0)	53 (25.5)	25 (26.9)	67 (19.7)
Blood transfusion	No	2253 (88.3)	190 (91.4)	86 (92.5)	290 (85.0)
	Yes	300 (11.8)	18 (8.7)	7 (7.5)	51 (15.0)
Non-surgical hospitalisation^†^	No	1065 (41.7)	122 (58.7)	52 (55.9)	
	Yes	1488 (58.3)	86 (41.4)	41 (44.1)	

*Among patients with VTE, 13.0% underwent lung cancer surgery after the diagnosis of cancer and before the diagnosis of VTE

^†^Non-surgical stay of more than 2 nights

**Table 4:** Risk factor analysis for venous thromboembolism (VTE) occurrence within 1 year after lung cancer diagnosis

		Univariate		Multivariate**	
		sHR (95% CI)	*p*-value	sHR (95% CI)	*p*-value
Age	< 55	–	–	–	–
	55–65	0.89 (0.61–1.28)	0.519	0.94 (0.64–1.38)	0.758
	65–75	0.66 (0.45–0.99)	0.042	0.78 (0.52–1.17)	0.232
	≥ 75	0.42 (0.27–0.65)	0.0001	0.65 (0.41–1.04)	0.071
Gender	Male	–	–	–	–
	Female	1.07 (0.80–1.43)	0.650	1.10 (0.82–1.48)	0.523
Metastasis		2.86 (2.12–3.86)	< 0.0001	1.83 (1.30–2.58)	0.0005
VTE concomitant with cancer diagnosis*		4.26 (1.67–10.88)	0.003	4.07 (1.57–10.52)	0.004
Lung cancer surgery		0.96 (0.64–1.44)	0.955		
Other surgery		2.05 (1.48–2.82)	< 0.0001	1.49 (1.06–2.09)	0.022
Chemotherapy		4.63 (3.45–6.23)	< 0.0001	3.44 (2.44–4.85)	< 0.0001
Bevacizumab		2.39 (1.29–4.42)	0.006		
Oral targeted therapy		1.19 (0.56–2.54)	0.655		
Radiotherapy		1.95 (1.41–2.71)	< 0.0001		
Blood transfusion		1.91 (1.16–3.15)	0.012		
Non-surgical hospitalization		1.53 (1.16–2.03)	0.003		

*Risk of recurrent VTE beyond 6 months after the first VTE

**Multivariate analysis adjusted for age and gender

**Table 5:** Risk factor analysis for PE occurrence within 1 year after lung cancer diagnosis

		Univariate		Multivariate**	
		sHR (95% CI)	*p*-value	sHR (95% CI)	*p*-value
Age	< 55	–	–	–	–
	55–65	0.82 (0.45–1.48)	0.503	0.88 (0.48–1.60)	0.665
	65–75	0.75 (0.40–1.37)	0.346	0.91 (0.49–1.70)	0.774
	≥ 75	0.72 (0.39–1.33)	0.294	1.08 (0.56–2.08)	0.828
Gender	Male	–	–	–	–
	Female	1.63 (1.08–2.47)	0.020	1.68 (1.11–2.54)	0.014
Metastasis		3.46 (2.18–5.50)	< 0.0001	2.51 (1.48–4.26)	0.0006
VTE concomitant with cancer diagnosis*		3.33 (0.80–13.97)	0.100		
Lung cancer surgery		0.78 (0.41–1.50)	0.456		
Other surgery		1.64 (0.98–2.75)	0.061		
Chemotherapy		3.97 (2.58–6.09)	< 0.0001	3.13 (1.88–5.20)	< 0.0001
Bevacizumab		1.39 (0.44–4.42)	0.577		
Oral targeted therapy		2.38 (1.02–5.53)	0.045		
Radiotherapy		2.10 (1.29–3.43)	0.003		
Blood transfusion		1.53 (0.70–3.34)	0.286		
Non-surgical hospitalization		1.74 (1.14–2.65)	0.011		

*Risk of recurrent VTE beyond 6 months after the first VTE

**Multivariate analysis adjusted for age and gender

**Table 6:** Treatments delivered during the 2 months preceding a major bleeding event after lung cancer diagnosis

		Total	Major bleeding type		
**N (%)**		**(*n* = 341)**	**Digestive**	**Intracranial**	**Other**
Total			55 (16.1)	22 (6.5)	264 (77.4)
**Anticoagulant or antiplatelet treatment**	**No**	**172 (50.4)**	**26 (47.3)**	**8 (36.4)**	**138 (52.3)**
	**Yes**	**169 (49.6)**	**29 (52.7)**	**14 (63.6)**	**126 (47.7)**
Anticoagulant therapy	No	263 (77.1)	40 (72.7)	16 (72.7)	207 (78.4)
	Yes	78 (22.9)	15 (27.3)	6 (27.3)	57 (21.6)
Antiplatelet therapy	No	225 (66.0)	37 (67.3)	11 (50.0)	177 (67.1)
	Yes	116 (34.0)	18 (32.7)	11 (50.0)	87 (32.9)
NSAID treatment	No	312 (91.5)	50 (90.9)	19 (86.4)	243 (92.1)
	Yes	29 (8.5)	5 (9.1)	3 (13.6)	21 (7.9)

**Table 7:** Risk factor analysis for major bleeding occurrence within 1 year after lung cancer diagnosis

		Univariate		Multivariate**	
		sHR (95% CI)	*p*-value	sHR (95% CI)	*p*-value
Age	< 65	–	–	–	–
	≥ 65	1.38 (1.11–1.71)	0.004	1.40 (1.11–1.76)	0.005
Gender	Male	–	–	–	–
	Female	0.68 (0.53–0.87)	0.002	0.73 (0.57–0.94)	0.016
Metastasis		1.58 (1.26–1.98)	< 0.0001	1.38 (1.08–1.78)	0.011
PE		2.88 (1.83–4.52)	< 0.0001	2.90 (1.85–4.54)	< 0.0001
DVT		1.53 (0.96–2.43)	0.074		
Anticoagulant therapy		1.91 (1.41–2.59)	< 0.0001	1.77 (1.30–2.43)	0.0003
Antiplatelet therapy		1.36 (1.09–1.71)	0.007	1.27 (1.01–1.60)	0.044
NSAID therapy		0.71 (0.49–1.03)	0.075		
Lung cancer surgery		1.79 (1.33–2.42)	0.0001	2.54 (1.88–3.45)	< 0.0001
Other surgery		1.68 (1.26–2.23)	0.0004	1.50 (1.11–2.02)	0.009
Chemotherapy		1.66 (1.30–2.13)	< 0.0001	1.54 (1.19–2.00)	0.001
Bevacizumab		1.04 (0.49–2.21)	0.919		
Oral targeted therapy		0.97 (0.47–1.97)	0.925		
Radiotherapy		1.80 (1.34–2.41)	0.0001	1.71 (1.25–2.34)	0.001

**Multivariate analysis adjusted for age and gender

Cancer is now an undoubted risk factor, with 15–20% of all VTE occurring in cancer patients [6]. There is a 7-fold increased risk of VTE in patients with cancer, and the risk is highest in the months following diagnosis [7]. The prevalence of VTE in cancer is estimated to be more than 2% in hospitalized patients [8]. Several risk factors have been described in cancer patients, including surgery, radiotherapy, chemotherapy and antiangiogenic treatments, metastatic stage, adenocarcinoma type, advanced stages of cancer, and hospitalization [6–12].

Lung cancer is increasingly frequent, and, in 2017, its estimated incidence in France was almost 50,000 new cases per year. It is the most common cause of death by cancer, with a 5-year survival rate of only 17% [13].

Lung cancer is one of the cancer types associated with the highest risk of developing VTE [7]. Hall et al. found an incidence of 6/100 person-years using the United States of America Medicare data [14], and Walker et al. found 39.2/1000 person-years using the United Kingdom health insurance database [15]. Though lung resection is an increasingly common treatment for lung cancer [16], this surgery increases the risk of VTE significantly. Platinum-based chemotherapies are another major treatment that appear to increase the risk of VTE [17].

VTE management in patients with cancer is further complicated by an increased risk of bleeding which is potentially exacerbated by anticoagulant therapies; about 10% of patients with cancer will present at least one bleeding event [18]. This can be explained by the tumour itself but can also be brought on by invasive surgery or chemo/radiotherapy-induced thrombocytopenia.

While the issues of VTE, major bleeding and survival in lung cancer patients have previously been studied in other countries, to the best of our knowledge, no study including both hospital and community treated VTE has been conducted in France.

### Objective

The primary objective of this study was to evaluate the impact of VTE and major bleeding events on one-year survival after a primary lung cancer diagnosis in France. The secondary objective was to identify significant risk factors for VTE and major bleeding for this population.

## Methods

### Data source

Our study is based on the *échantillon généraliste des bénéficiaires* (EGB), a representative cross-sectional random sample (including hospital and out-of-hospital care) extracted from the total population database recorded by the French national insurance system (SNIIRAM - *Système National d’Information Interrégime de l’Assurance Maladie*) since 2004*.* The aim of the EGB database is to follow beneficiaries’ health care consumption over a period of 20 years. It was created using a systematic sampling method (1/97) based on the two-digit control key of beneficiaries’ national identification numbers, and it includes both current year reimbursement recipients and non-recipients. It is available to accredited researchers and contains the following individual, exhaustive and linkable but anonymous data [19]:

i) patients’ characteristics such as sex, age, date of death;
ii) the hospital discharge abstract database (*Programme de Médicalisation des Systèmes d’Informations* [PMSI]), which collects main and associated diagnoses encoded using the International Classification of Diseases 10th revision (ICD-10), and procedures performed during hospital stays (in all public and private hospitals), using the French common classification system for medical procedures (*Classification Commune des Actes Médicaux* [CCAM]);
iii) the reimbursement data for out-of-hospital care (consultations, procedures, drugs);
iv) the codes for long-term illnesses (*Affection Longue Durée* [ALD]), which provide patient coverage.

Various control procedures are regularly conducted to ensure the quality of these data. The reliability of the SNIIRAM, which initially included only the hospital database [16, 20–25] but more recently the whole database [26–28], has been established in recent studies.

### Study population

We conducted a nationwide, population-based, retrospective study based on randomly sampled individuals older than 18 years old who were diagnosed with primary lung cancer in France from the 1st of January 2009 to the 31st December 2015. Inclusion criteria were a malignant tumour of the lungs or bronchus (ICD-10 code: C34) selected either in the ALD or PMSI hospital database.

Exclusion criteria were non-primary lung cancer defined as a diagnosis in the PMSI or an attachment to an ALD for an ongoing malignant tumour other than lung, bronchus and brain tumour within 5 years before lung cancer diagnosis.

To determine the date of lung cancer diagnosis, we retained the first date between the first ALD registration and the first hospital stay with a diagnosis of lung cancer.

### Assessment of events

Follow-up started the day after the cancer diagnosis and was censored 1 year after. The availability of all data up to the 31st December 2016 made it possible to obtain a 1-year follow-up for all patients included. PE, DVT and bleeding events diagnosed during a hospital stay were identified from the associated ICD-10 codes in the PMSI database as a primary, related or associated diagnosis.

VTE diagnosed in ambulatory settings were identified in the EGB from the performance of investigations for VTE and by the delivery of a curative dose of anticoagulants within 8 days. The following investigations were identified: Doppler ultrasonography, pulmonary ventilation perfusion scintigraphy and CT pulmonary angiography. Data relative to the delivery of anticoagulants (heparin and oral anticoagulants) were collected. If an anticoagulant was delivered in the 72 h before the investigation exam, another delivery was required in the 2 following months. Therefore, patients for whom treatment was started before the investigation and then discontinued after a negative result were not misclassified as having VTE. We assumed that the events found only in an ambulatory setting were DVT seeing as a diagnosis of PE is more likely to be found in hospitalization data. We distinguished curative from preventive doses according to the quantities delivered, the time between two deliveries, duration of the treatment and national guidelines for VTE management. Major bleeding events were identified according the ICD-10 codes selected in the NACORA-BR study [29], which also analysed major bleeding events in France using a similar database. All coded bleeding events were considered to be major because the presence of a code meant that patient management required hospitalization. Intracranial and digestive bleeding events were identified. We classified bleeding events as “other” if they were not digestive or intracranial (such as gynaecological or ocular), or if the ICD-10 code did not specify the bleeding area. The PMSI does not provide precise diagnosis dates, so the date of admission was considered to be the diagnosis date.

Events were analysed by an adjudication committee of three experts, an angiologist, a pneumologist and a pharmacist, who independently evaluated the plausibility of the occurrence of an event given the collected data.

### Statistical analysis

Descriptive analyses were used to establish the population characteristics and to estimate the global frequency of VTE and major bleeding. Median survival with or without VTE or major bleeding were estimated using the Kaplan-Meier method.

A survival analysis was performed using Cox’s proportional hazard model to estimate the effect of VTE and major bleeding on one-year survival, adjusted for the other covariates. Treatment performance was introduced in the models as time-dependent covariates in order to take into account the time between the treatment and the event.

The Fine and Gray survival model was used to analyse VTE and major bleeding risk factors since death can be considered as a competitive risk with both of these events. Subdistribution hazard ratios (sHR) were estimated with this model.

Covariates included age, gender, Charlson’s comorbidity index (calculated with a previously validated algorithm [30] using information collected during the year preceding the inclusion), and concomitant diagnoses of VTE and cancer (defined as VTE occurring within 2 months before the date of cancer diagnosis or on the same date).

We also collected data concerning the presence of metastases, hospitalization and treatment (including chemotherapy, surgery (particularly for lung cancer), transfusion), bevacizumab, oral lung cancer targeted therapies and radiotherapy. Deliveries of anticoagulants, antiplatelet therapies and nonsteroidal anti-inflammatory drugs (NSAIDs) were also collected. All of the ICD-10, CCAM, CIP (Presentation Identification Code) and UCD (Common Dispensing Unit) codes used to collect the data are available online as supplementary material.

Similar analyses were performed specifically for PE occurrence and on the subgroup of patients presenting metastases.

For each survival analysis, all variables identified as significantly related to the occurrence of the event in a univariate analysis (using the Cox or Fine and Gray model) with a probability level of 0.20 were analysed in the multivariate regression model, except for age and gender which were systematically included. Proportional hazards and interactions were checked for each covariate. A backward selection of covariates was performed with a probability level of 0.05. Hazard ratios (HR) for Cox models or subdistribution hazard ratios (sHR) for Fine and Gray models are presented with 95% confidence intervals (CI). All analyses were performed with SAS software (SAS Institute Inc., Version 9.4, Cary, NC).

### Ethical and regulatory aspects

Written consent was not needed for this non-interventional retrospective study. Access to the data was granted on December 1st, 2016 (registration number 221) from the health data institute (*Institut national des données de santé*). The EGB database use was approved by the French national data protection agency (CNIL), and this study was conducted in accordance with the Declaration of Helsinki.

## Results

A total of 2553 patients were included in the study. A VTE episode was identified in the year following the diagnosis of lung cancer for 208 (8.2%) patients and a major bleeding event was identified for 341 (13.4%) patients. Among the patients who presented with VTE, 93 presented with PE and 115 with DVT alone. A VTE event concomitant with cancer diagnosis was found for 64 patients (2.5%) and metastasis were identified in 1535 (60.1%) patients. Among the patients who presented with a major bleeding event, 55 had digestive bleeding, 22 intracranial bleeding and the others had other sites of bleeding. More than 45% of patients in this study (1175) died during follow-up.

Patients were aged 23 to 100 years old with a mean age of 67 years. Table 1 presents the patients’ demographic and clinical characteristics as well as the frequency of VTE and major bleeding according to these characteristics.

### Primary objective: survival

Close to half of the patients who presented with VTE (104) died during follow-up. Among these patients, 56 presented with PE (accounting for 60.2% of the 93 patients with PE) and 48 presented with DVT alone (accounting for 41.7% of the 115 patients with DVT alone). The median survival time was 365.5 days for patients who presented with VTE and 439 days for those who did not. The mean time from PE to death was 105 ± 84 days, and 25% of these deaths occurred during the month following PE. The mean time from DVT alone to death was 124 ± 71, and 4% of these deaths occurred during the month following DVT. The median survival time was 326 days for patients who presented with major bleeding and 457 days for those who did not. Among the patients who presented with a major bleeding event, 186 died during follow-up (54.5%). The mean time from the major bleeding event to death was 89 ± 77 days, and 25% of these deaths occurred during the month following major bleeding. The median survival time was 355.5 days for patients who presented with VTE and major bleeding and 427 days for those who presented with neither VTE nor major bleeding. As shown in Table 2, VTE occurrence was associated with a significantly increased risk of death (HR = 1.53 [1.20–1.95] for PE and HR = 1.26 [1.01–1.57] for DVT), as was major bleeding occurrence (HR = 1.81 [1.54–2.12]).

### Secondary objective: risk factors for VTE and major bleeding

VTE treatment was performed in an ambulatory setting for 49 patients (23.5%). More than 75% of VTE events occurred during the first 6 months following cancer diagnosis. A major bleeding event was found for 19 (9.1%) patients during the month following VTE. VTE occurred in 2 (0.6%) patients during the month following a major bleeding event.

Major treatments received during follow-up and before VTE, PE or major bleeding occurrence are described in Table 3.

### VTE risk factors

Concomitant VTE and cancer diagnosis was associated with an increased risk of VTE recurrence beyond 6 months after the first VTE event (sHR = 4.07, 95% CI [1.57–10.52]). Although bevacizumab administration, radiotherapy, blood transfusion and non-surgical hospitalization were associated with VTE occurrence in the bivariate analysis, the only covariates remaining in the multivariable model were metastases (sHR = 1.83, 95% CI [1.30–2.58]), chemotherapy (sHR = 3.44, 95% CI [2.44–4.85]) and surgery other than lung cancer resection (sHR = 1.49, 95% CI [1.06–2.09]), as shown in Table 4. Lung cancer surgery and oral targeted therapies were not associated with an increased risk of VTE.

### PE risk factors

Table 5 shows quite similar results for PE occurrence, but female gender was significantly associated with PE in the multivariable model (sHR = 1.68 [1.11–2.54]) whereas surgery and concomitant VTE and cancer diagnoses were no longer significant.

### Major bleeding risk factors

The delivery of anticoagulants, antiplatelet therapies and NSAIDs before a major bleeding event are detailed in Table 6. Among patients presenting major bleeding, 78 (22.9%) had an anticoagulant treatment delivered during the 2 months preceding the event, 116 (34%) had an antiplatelet therapy delivered and 169 (49.6%) had either an anticoagulant or an antiplatelet therapy delivered. An NSAID treatment was delivered to 29 (8.5%) patients before a major bleeding event. The rate of major bleeding episodes in patients with VTE was 18.3% versus 12.9% in patients without VTE. Patients over 65 years old had an increased risk of bleeding (sHR = 1.40 [1.11–1.76]), as shown in Table 7, but women had a lower risk than men (sHR = 0.73 [0.57–0.94]). There were also highly significant increases in bleeding risk following a PE event (sHR = 2.90 [1.85–4.54]) and lung cancer surgery (sHR = 2.54 [1.88–3.45]). Chemotherapy, radiotherapy, surgery and metastasis were also found to be associated with the risk of major bleeding.

Subgroup analyses with patients presenting metastases provided similar results.

## Discussion

### Survival

In this study, the rate of death following VTE was high. This was especially true for patients who presented with PE since 60% of them died during follow-up. We also observed an elevated rate of death following major bleeding considering that 55% of these patients died within the follow-up period. This rate is consistent with the findings of Chouaid et al. in the TERRITOIRE study [31] in which the risk of death was significantly increased following PE and major bleeding events.

### VTE frequency and risk factors

We report a high frequency (8%) of VTE after lung cancer diagnosis in France, with an increased risk of death after VTE occurrence. This result is consistent with other studies such as the FRAGMATIC trial, where 9.7% of newly diagnosed patients with no low-molecular-weight heparin prophylaxis presented with VTE [32]. While VTE is a well-established prognosis factor in cancer patients [33], we demonstrate that clinical and therapeutic factors such as chemotherapy, surgery and the presence of metastases are related to VTE occurrence. Concomitant VTE and cancer diagnoses are associated with an increased risk of VTE after cancer diagnosis, with a sHR of 4.07 [1.57–10.52]. This result calls into question the effectiveness of VTE management for these patients. As suggested by Piran et al. [34], treatment and surveillance protocols may not be effective enough to prevent VTE recurrence. Patient adherence to treatment is another potential issue. In future studies, it would be interesting to analyse the use of anticoagulation therapies (type, duration) when prophylaxis is recommended for these patients.

Chemotherapy was found to be majorly associated with the risk of VTE (3.44 sHR), which is consistent with previously published studies [35]. According to the literature, this risk is increased particularly during chemotherapy and for a month after discontinuation [36, 37]. The BIOTEL study showed that 15% of lung cancer patients presented with VTE within 6 months after initiating chemotherapy [38], Connolly et al. found 13.9% [37], and Khorana et al. found 12.6% [17]. More recently, Rupa-Matysek et al. reported that 16.9% of patients undergoing chemotherapy developed VTE [39]. Currently, some international guidelines suggest the use of primary thromboprophylaxis for metastatic lung cancer patients with low bleeding risk receiving chemotherapy [40].

Lung cancer surgery had a protective effect in the bivariate analysis, which is likely explained by the fact that this surgery is reserved for patients with a better prognosis. Only non-lung-cancer surgeries had a significant effect on VTE occurrence in the multivariable model (1.49 sHR). Surgery is a well-known risk factor for VTE, and its association with the thrombogenic state induced by cancer can explain part of this risk. However, it is not clear whether prophylaxis guidelines were properly followed for these patients.

Surprisingly, female gender seemed to be associated with a higher risk of PE. Women of childbearing age have been found to present a higher risk of VTE than men [5], which was explained by hormonal factors, but most of the women included in our study were no longer of childbearing age.

Bevacizumab was not significantly associated with events in the multivariable model. As all patients receiving bevacizumab were also undergoing chemotherapy, the effect of the two treatment regimens cannot be distinguished. However, when chemotherapy was not integrated in the multivariable models, bevacizumab was not significantly associated with VTE occurrence.

Non-surgical hospitalization is a major risk factor for VTE in the general population as it induces immobilization, but it does not appear to increase significantly the risk of VTE in lung cancer patients. These patients are more likely to be hospitalized and are exposed to other more significant risk factors which probably lessen the overall effect of hospitalization.

### Major bleeding frequency and risk factors

We also report frequent major bleeding events (13%) after a lung cancer diagnosis. In patients presenting with major bleeding, in the two previous month more than 22% had taken anticoagulant therapy and more than 30% antiplatelet therapy. It is interesting to note that half of bleeding events were seemingly not related to a pharmacological factor. NSAIDs were delivered to 8% of our patient population before the event, but no information on self-medication is available in the database. The rate of major bleeding episodes is higher for patients with VTE (18.3%) than for those without (12.9%). However, these rates are to interpret cautiously since follow-up started on the date of the cancer diagnosis (the primary objective being survival) and we only had information on the first bleeding event during follow-up. Major bleeding events may have occurred before the episode of VTE.

The risk of major bleeding was higher for patients over 65 years old and for men, even though gynaecologic bleeding was included. PE was identified as a significant risk factor for major bleeding, which can be explained by the addition of an anticoagulant therapy. An increased risk of major bleeding was found for patients taking anticoagulant or antiplatelet therapy, but not NSAIDs. The risk was also significantly increased after surgery, especially after lung cancer resection, which probably reflects the invasiveness of the procedure. Chemotherapy also seems to be associated with an increased risk of major bleeding, which could be explained by the haematological damage induced. Bevacizumab did not increase the risk of major bleeding.

### Study strengths and limitations

This is the first French study on VTE following cancer diagnosis which assesses the risk of various cancer treatments. One of its great strengths resides in the large number of patients included in a real-life setting. According to the National Institute of Cancer, 37,000 cases-year of lung cancer were diagnosed in France (2012 data) [41]. Therefore, about 259,000 cases of lung cancer should have been diagnosed in the whole population during the 7-year study period. Since we would expect to see about 2670 cases of lung cancer in a random sample like ours (1/97th of the whole population), our population of 2553 cases is consistent, which means that the present study can draw relevant conclusions on a national level. Another strength of this study is that we included VTE treated in an ambulatory setting even though cases were deduced indirectly. This is unlike most studies which only include patients treated in hospital, thus minimizing the extent of this issue. VTE treated in ambulatory care accounts for more than 20% of all VTE events in our study. Moreover, we introduced time-dependent covariates in the models. This allowed us to take into account the time between the treatment and the event and to estimate their effect more precisely by giving the appropriate weight to events occurring shortly after the treatment of interest.

The main limitation of this study is that the cause of death is not reported in the EGB, so we were only able to analyse overall survival. Some patients may have died from VTE, but this information was not assessable. However, real-life overall survival is still a clinically relevant indicator. We used a Fine and Gray model to analyse this competing risk setup (VTE and death). Further studies with access to the cause of death are needed to estimate the frequency of deaths that are a direct result of VTE events.

There is also an issue regarding the diagnosis dates provided by this database. However, the diagnosis date for cancer rarely marks the actual beginning of the disease, so the slight inaccuracy of these dates may not be clinically relevant. We cannot exclude that some of the dates were reported later than the actual diagnosis date, but the effect should be negligible considering the large size of the cohort. Moreover, we were able to study the chronology between events by introducing time-dependent variables in the model. Even though dates are imprecise, this allowed us to correctly qualify the exposure to each risk before the event for every patient, and this should not bias the results.

Another limitation is that some known risk factors for VTE such as cancer type, stage and mutational status were not available in this database. Other prognosis factors that are used to calculate a prognosis score validated in lung cancer patients were not available either, including smoking status, respiratory comorbidities and weight loss [42]. However, we did adjust for the presence of metastases in our model, which appears to be a major risk factor according to the literature.

It is interesting to notice the consistency of the results when we analysed the subgroup of patients with metastases. Even though the information concerning the presence of metastases may not be complete in the database, we can consider it reliable when available. An indication bias can be questioned seeing as bevacizumab, which is known to increase VTE and bleeding risk, was not found to be a risk factor in our study. This could be due to a selection of lower-risk patients for bevacizumab treatment. Furthermore, biological data such as platelet, hemoglobin and leukocyte levels were not available.

The chemotherapy agents used were not available in the database either, which is unfortunate because they appear to be predictive factors for VTE and major bleeding events. Lastly, we were only able to include patients up to 2015 to ensure a one-year follow-up with the data available. Given the rapid changes in lung cancer management over the last few years, analyses on more recent data could make a difference in these results.

## Conclusion

This is the first study to explore both VTE and major bleeding in patients diagnosed with lung cancer in France. We found a high frequency of VTE and an even higher frequency of major bleeding following a lung cancer diagnosis. As both VTE and major bleeding were shown to be significantly associated with decreased survival, and most major bleeding events were seemingly not related to a pharmacological factor, we may hypothesize that VTE and major bleeding could be considered independently as indicators of the worsening of lung cancer.

## Supplementary information

**Additional file 1.**

## Data Availability

Our study is based on the EGB random sample (including hospital and out-of-hospital care) extracted from the total population database recorded by the National Insurance system and made available (1/97th of the whole population) to accredited researchers that contains individual, exhaustive and linkable but anonymous data. We are not allowed to transmit these data.

## Abbreviations

VTE: Venous thromboembolism
DVT: deep vein thrombosis
PE: pulmonary embolism
EGB: échantillon généraliste des bénéficiaires
SNIIRAM: Système National d’Information Interrégime de l’Assurance Maladie
PMSI: Programme de Médicalisation des Systèmes d’Informations
ICD-10: International Classification of Diseases 10th revision
CCAM: Classification Commune des Actes Médicaux
ALD: Affection Longue Durée
CT: computed tomography
sHR: Subdistribution hazard ratios
NSAIDs: nonsteroidal anti-inflammatory drugs
UCD: Common Dispensing Unit
HR: hazard ratios
CI: confidence intervals
INDS: Health data institute (Institut national des données de santé)
CNIL: French national data protection agency
