# Supplementary material for: Could venous thromboembolism and major bleeding be indicators of lung cancer mortality? A nationwide database study
Source: BMC Cancer. 2020 May 24;20:461. doi: 10.1186/s12885-020-06930-1 (PMC7245783; doi:10.1186/s12885-020-06930-1)
Supplement: Supplementary file 1 — Additional file 1. [file 12885_2020_6930_MOESM1_ESM.docx]

Supplementary material:

**Common classification of medical acts codes (CCAM)**

**VTE investigations:**

**Doppler Ultrasonography:**

| EFQM001 | Doppler ultrasonography of the upper limb veins |
| --- | --- |
| EJQM003 | Doppler ultrasonography of the lower limb and iliac veins for deep vein thrombosis research |

**Pulmonary perfusion scintigraphy:**

| GFQL002 | Ventilation and perfusion pulmonary PET-scan |
| --- | --- |
| GFQL005 | Perfusion pulmonary PET-scan |
| GFQL006 | Ventilation and perfusion pulmonary scan |
| GFQL007 | Perfusion pulmonary scan |

**Thoracic angiography:**

| ECQH010 | Thoracic angiography |
| --- | --- |
| ECQH011 | Thoracic, abdominal and pelvic angiography |

**Lung cancer surgery:**

| GEFA004 | Resection-anastomosis of the tracheal bifurcation by thoracotomy |
| --- | --- |
| GEFA011 | Bronchial resection-anastomosis by thoracotomy |
| GFFA001 | Pneumonectomy with complete pleural excision by thoracotomy |
| GFFA002 | Pneumonectomy with resection and prosthetic replacement of the superior vena cava |
| GFFA004 | Pulmonary lobectomy with resection-anastomosis or bronchus reimplantation |
| GFFA007 | Pneumonectomy with organ and/or nearby structure resection, by thoracotomy |
| GFFA009 | Pulmonary lobectomy, by thoracotomy with thoracoscopic preparation |
| GFFA010 | Pulmonary bilobectomy with thoracic wall resection by thoracotomy |
| GFFA011 | Pneumonectomy with resection-anastomosis of the tracheal bifurcation by thoracotomy |
| GFFA013 | Pulmonary bilobectomy, by thoracotomy |
| GFFA017 | Single partial non-anatomic pulmonary excision by thoracotomy |
| GFFA018 | Pulmonary bilobectomy by thoracotomy |
| GFFA019 | Excision of the remaining pulmonary lobe by thoracotomy |
| GFFA021 | Multiple partial non-anatomic pulmonary excision by thoracotomy |
| GFFA022 | Pulmonary lobectomy with organ and/or nearby structure resection |
| GFFA024 | pneumonectomy by thoracotomy |
| GFFA025 | Pneumonectomy with thoracic wall resection, by thoracotomy. |
| GFFA026 | Pulmonary lobectomy with resection-anastomosis of the tracheal bifurcation, by thoracotomy |
| GFFA027 | Pulmonary lobectomy with thoracic wall resection, by thoracotomy |
| GFFA028 | Pneumonectomy with resection-anastomosis of the superior vena cava. |
| GFFA029 | Single or multiple pulmonary segmentectomy by thoracotomy. |
| GFFA034 | Pulmonary bilobectomy with organ and/or nearby structure resection |

**Radiotherapy:**

| AANL001-002 | Encephalic radiation |
| --- | --- |
| AZNL001 | Craniospinal radiation |
| BHNL001 | Ocular-globe transscleral brachytherapy |
| EZNF900 | Intravascular radiation |
| FENL001 | Internal radiation for polycythemia, by intravenous injection of Phosphorus 32 |
| JGNL001 | Prostate brachytherapy by permanent Iodine 125 insertion |
| JKNL001-006 | Uterovaginal intracavitary brachytherapy |
| JLNL001-008 | Vaginal intracavitary brachytherapy |
| KCNL003-004 | Internal radiation of the thyroid gland by iodine 131 |
| PANL001 | Internal radiation of a bone injury by intravenous injection of a pharmacologic radioisotope |
| QZNL001 | Total skin radiation |
| YYYY016  YYYY021  YYYY023 | Contact radiation for malignant tumor |
| YYYY045-050  YYYY099  YYYY101  YYYY136  YYYY152  YYYY197  YYYY211  YYYY244  YYYY299  YYYY301-305  YYYY307  YYYY310  YYYY312-316  YYYY320  YYYY323-327  YYYY331  YYYY334-338  YYYY343  YYYY345  YYYY348-349  YYYY356-360  YYYY365  YYYY367-371  YYYY377  YYYY379-383  YYYY387  YYYY390-393  YYYY398  YYYY450-451  YYYY457-460  YYYY468-471  YYYY479-481  YYYY491-493  YYYY497  YYYY500  YYYY511  YYYY520  YYYY522  YYYY533  YYYY544 | Fixed field radiation |
| YYYY051-056  YYYY122  YYYY141  YYYY175  YYYY223  YYYY256  YYYY267  YYYY306  YYYY555  YYYY566  YYYY577  YYYY588  YYYY599 | Cyclotherapy |
| ZANL001 | Intracranial external radiation |
| ZZNA002 | Intraoperative external radiation |
| ZZNL001-006  ZZNL009  ZZNL011-015  ZZNL017-018 | Interstitial brachytherapy |
| ZZNL007-008  ZZNL010  ZZNL019 | Intraluminal brachytherapy |
| ZZNL016 | Internal radiation by transcutaneous intravenous injection of a pharmacologic radioisotope |
| ZZNL020-021  ZZNL023-028  ZZNL030-031  ZZNL033-034  ZZNL036-037  ZZNL039-040  ZZNL042-043  ZZNL045-065  ZZNL900  ZZNL902-906 | External radiation |
| ZZNL066 | Endocavitary contact radiation |

**Medication’s presentation identification codes (CIP)**

**Anticoagulants**

**« Curative » doses:**

| 3400955670360 | CALCIPARINE 12500UI/0,5ML SOL INJ A (heparin calcium) |
| --- | --- |
| 3400930167632 | CALCIPARINE 20000UI/0,8ML SOL INJ S (heparin calcium) |
| 3400936468955 | LOVENOX 10000UI AXa/1ML INJ SER +S (enoxaparin sodium) |
| 3400936469037 | LOVENOX 6000UI AXa/0,6ML INJ SER +S (enoxaparin sodium) |
| 3400936469266 | LOVENOX 6000UI AXa/0,6ML INJ SER +S (enoxaparin sodium) |
| 3400936469327 | LOVENOX 8000UI AXa/0,8ML INJ SER +S (enoxaparin sodium) |
| 3400936469495 | LOVENOX 8000UI AXa/0,8ML INJ SER +S (enoxaparin sodium) |
| 3400933972394 | INNOHEP 10000UI AXa/0,5ML INJ SER (tinzaparin sodium) |
| 3400933972516 | INNOHEP 10000UI AXa/0,5ML INJ SER (tinzaparin sodium) |
| 3400933972684 | INNOHEP 14000UI AXa/0,7ML INJ SER (tinzaparin sodium) |
| 3400933972806 | INNOHEP 14000UI AXa/0,7ML INJ SER (tinzaparin sodium) |
| 3400933973117 | INNOHEP 18000UI AXa/0,9ML INJ SER (tinzaparin sodium) |
| 3400933972974 | INNOHEP 18000UI AXa/0,9ML INJ SER (tinzaparin sodium) |
| 3400933246549 | FRAXIPARINE 7600UI AXa/0,8ML SOL INJ (nadroparin calcium) |
| 3400933488963 | FRAXIPARINE 7600UI AXa/0,8ML SOL INJ (nadroparin calcium) |
| 3400932783229 | FRAXIPARINE 9500UI AXa/1ML SOL INJ (nadroparin calcium) |
| 3400934732881 | FRAXODI 11400UI AXa/0,6ML SOL INJ (nadroparin calcium) |
| 3400934733024 | FRAXODI 11400UI AXa/0,6ML SOL INJ (nadroparin calcium) |
| 3400934733192 | FRAXODI 15200UI AXa/0,8ML SOL INJ (nadroparin calcium) |
| 3400934733314 | FRAXODI 15200UI AXa/0,8ML SOL INJ (nadroparin calcium) |
| 3400934733482 | FRAXODI 19000UI AXa/1ML SOL INJ (nadroparin calcium) |
| 3400934733604 | FRAXODI 19000UI AXa/1ML SOL INJ (nadroparin calcium) |
| 3400949250196 | FRAGMINE 10000UI AXa/0,4ML INJ SER (dalteparin sodium) |
| 3400933730840 | FRAGMINE 10000UI AXa/1ML SOL INJ SER (dalteparin sodium) |
| 3400934213052 | FRAGMINE 10000UI AXa/1ML SOL INJ SER (dalteparin sodium) |
| 3400939743233 | FRAGMINE 12500UI AXa/0,5ML SOL INJ (dalteparin sodium) |
| 3400939743462 | FRAGMINE 15000UI AXa/0,6ML SOL INJ (dalteparin sodium) |
| 3400939743523 | FRAGMINE 18000UI AXa/0,72ML SOL INJ (dalteparin sodium) |
| 3400939743004 | FRAGMINE 7500UI AXa/0,3ML SOL INJ (dalteparin sodium) |
| 3400933730611 | FRAGMINE 7500UI AXa/0,75ML SOL INJ (dalteparin sodium) |
| 3400934212970 | FRAGMINE 7500UI AXa/0,75ML SOL INJ (dalteparin sodium) |
| 3400936565340 | ARIXTRA 10MG/0,8ML SOL INJ SER (fondaparinux sodium) |
| 3400936565579 | ARIXTRA 10MG/0,8ML SOL INJ SER (fondaparinux sodium) |
| 3400936564749 | ARIXTRA 5MG/0,4ML SOL INJ SER (fondaparinux sodium) |
| 3400936564978 | ARIXTRA 5MG/0,4ML SOL INJ SER (fondaparinux sodium) |
| 3400936565050 | ARIXTRA 7,5MG/0,6ML SOL INJ SER (fondaparinux sodium) |
| 3400936565289 | ARIXTRA 7,5MG/0,6ML SOL INJ SER (fondaparinux sodium) |

**« Curative or preventive » doses:**

| 3400932782857 | FRAXIPARINE 5700UI AXa/0,6ML SOL INJ (nadroparin calcium) |
| --- | --- |
| 3400933487614 | FRAXIPARINE 5700UI AXa/0,6ML SOL INJ (nadroparin calcium) |
| 3400933484132 | PREVISCAN 20MG CPR (fluindione) |
| 3400930264546 | COUMADINE 2MG CPR (warfarin sodium) |
| 3400935693099 | COUMADINE 5MG CPR (warfarin sodium) |
| 3400933662912 | MINISINTROM 1MG CPR (acenocoumarol) |
| 3400933564308 | SINTROM 4MG CPR (acenocoumarol) |
| 3400921922516 | XARELTO 15MG CPR (rivaroxaban) |
| 3400921922684 | XARELTO 15MG CPR (rivaroxaban) |
| 3400921922745 | XARELTO 15MG CPR (rivaroxaban) |
| 3400921923056 | XARELTO 20MG CPR (rivaroxaban) |
| 3400938526080 | PRADAXA 110MG GELULE (dabigatran etexilate) |
| 3400938526141 | PRADAXA 110MG GELULE (dabigatran etexilate) |
| 3400938526202 | PRADAXA 110MG GELULE (dabigatran etexilate) |
| 3400941945380 | PRADAXA 150MG GELULE (dabigatran etexilate) |
| 3400926784102 | ELIQUIS 5MG CPR (apixaban) |

**« Preventive » doses:**

| 3400931878612 | CALCIPARINE 5000UI/0,2ML SOL INJ SER (heparin calcium) |
| --- | --- |
| 3400931878551 | CALCIPARINE 7500UI/0,3ML SOL INJ SER (heparin calcium) |
| 3400936468375 | LOVENOX 2000UI AXa/0,2ML INJ SER +S (enoxaparin sodium) |
| 3400936468436 | LOVENOX 2000UI AXa/0,2ML INJ SER +S (enoxaparin sodium) |
| 3400936468665 | LOVENOX 4000UI AXa/0,4ML INJ SER +S (enoxaparin sodium) |
| 3400936468726 | LOVENOX 4000UI AXa/0,4ML INJ SER +S (enoxaparin sodium) |
| 3400933433093 | INNOHEP 2500UI AXa/0,25ML INJ SER (tinzaparin sodium) |
| 3400933582456 | INNOHEP 2500UI AXa/0,25ML INJ SER (tinzaparin sodium) |
| 3400933433215 | INNOHEP 3500UI AXa/0,35ML INJ SER (tinzaparin sodium) |
| 3400933582166 | INNOHEP 3500UI AXa/0,35ML INJ SER (tinzaparin sodium) |
| 3400934216992 | INNOHEP 4500UI AXa/0,45ML INJ SER (tinzaparin sodium) |
| 3400934217074 | INNOHEP 4500UI AXa/0,45ML INJ SER (tinzaparin sodium) |
| 3400932782567 | FRAXIPARINE 2850UI AXa/0,3ML SOL INJ (nadroparin calcium) |
| 3400933389178 | FRAXIPARINE 2850UI AXa/0,3ML SOL INJ (nadroparin calcium) |
| 3400933010966 | FRAGMINE 2500UI AXa/0,2ML SOL INJ (dalteparin sodium) |
| 3400933530129 | FRAGMINE 2500UI AXa/0,2ML SOL INJ (dalteparin sodium) |
| 3400933010737 | FRAGMINE 5000UI AXa/0,2ML SOL INJ (dalteparin sodium) |
| 3400933529819 | FRAGMINE 5000UI AXa/0,2ML SOL INJ (dalteparin sodium) |
| 3400935922540 | ARIXTRA 2,5MG/0,5ML SOL INJ SER (fondaparinux sodium) |
| 3400935922601 | ARIXTRA 2,5MG/0,5ML SOL INJ SER (fondaparinux sodium) |
| 3400956361977 | ARIXTRA 2,5MG/0,5ML SOL INJ SER (fondaparinux sodium) |
| 3400938838107 | XARELTO 10MG CPR (rivaroxaban) |
| 3400938838275 | XARELTO 10MG CPR (rivaroxaban) |
| 3400938838336 | XARELTO 10MG CPR (rivaroxaban) |
| 3400949506781 | XARELTO 10MG CPR ADP MWI (rivaroxaban) |
| 3400938525540 | PRADAXA 75MG GELULE (dabigatran etexilate) |
| 3400938525601 | PRADAXA 75MG GELULE (dabigatran etexilate) |
| 3400938525779 | PRADAXA 75MG GELULE (dabigatran etexilate) |
| 3400941945441 | ELIQUIS 2,5MG CPR (apixaban) |
| 3400941945502 | ELIQUIS 2,5MG CPR (apixaban) |
| 3400941945670 | ELIQUIS 2,5MG CPR (apixaban) |

**Oral lung cancer targeted therapies:**

| 3400936923461 | TARCEVA 100MG CPR (erlotinib) |
| --- | --- |
| 3400936923522 | TARCEVA 150MG CPR (erlotinib) |
| 3400936923232 | TARCEVA 25MG CPR (erlotinib) |
| 3400939595078 | IRESSA 250MG CPR (gefitinib) |
| 3400893987834 | GIOTRIF 20MG CPR (afatinib) |
| 3400927565700 | GIOTRIF 30MG CPR (afatinib) |
| 3400927565878 | GIOTRIF 40MG CPR (afatinib) |
| 3400927565939 | GIOTRIF 50MG CPR (afatinib) |
| 3400926762568 | XALKORI 200MG GELULE (crizotinib) |
| 3400926762797 | XALKORI 250MG GELULE (crizotinib) |
| 3400930015698 | ZYKADIA 150MG GELULE (ceritinib) |

**Medication’s common delivery unit codes (UCD)**

**Bevacizumab:**

| 3400892611044 | AVASTIN 25MG/ML SOL INJ FL 4ML (bevacizumab) |
| --- | --- |
| 3400892611105 | AVASTIN 25MG/ML SOL INJ FL 16ML (bevacizumab) |

**Algorithms for ambulatory treated VTE identification**

D0: day of VTE investigation

| **VTE** |
| --- |
| “Curative” dose delivered from D1 to D8 |
| “Curative” dose delivered from D-3 to D0  + “curative” dose from D9 to D60 |
| “Curative” dose delivered on D0  + “Curative or preventive” dose delivered from D0 to D20  + “Curative or preventive” dose delivered from D21 to D60 |
| >28 Rivaroxaban 15mg units delivered from D1 to D8 |
| **No VTE** |
| No “Curative” or “Curative or preventive” dose of anticoagulant delivered from D-3 to D8 |
| “Curative” or “Curative or preventive” dose of anticoagulant delivered from D-3 to D0  + No “Curative” or “Curative or preventive” dose of anticoagulant delivered from D1 to D60 |
| “Curative or preventive” dose of anticoagulant delivered from D-3 to D8  + only deliveries of the same anticoagulant from D-60 to D-4 |
| Only deliveries of Dabigatran, Rivaroxaban 20mg or Vitamin K agonists from D-3 to D+60  + investigation performed in an ambulatory setting |
